# Variability in white matter structure relates to hallucination proneness

**DOI:** 10.1016/j.nicl.2024.103643

**Published:** 2024-07-20

**Authors:** Joseph F. Johnson, Michael Schwartze, Michel Belyk, Ana P. Pinheiro, Sonja A. Kotz

**Affiliations:** a Université Libre de Bruxelles, Center for Research in Cognition & Neurosciences, Bruxelles, Belgium; b University of Maastricht, Department of Neuropsychology and Psychopharmacology, Maastricht, The Netherlands; c Edge Hill University, Department of Psychology, Ormskirk, United Kingdom; d Faculdade de Psicologia, Universidade de Lisboa, Lisboa, Portugal; e Max Planck Institute for Human and Cognitive Sciences, Department of Neuropsychology, Leipzig, Germany

**Keywords:** Diffusion tensor imaging, Hallucination, Psychosis-like experience, White matter, Cerebellum

## Abstract

Hallucinations are a prominent transdiagnostic psychiatric symptom but are also prevalent in individuals who do not require clinical care. Moreover, persistent psychosis-like experience in otherwise healthy individuals may be related to an increased risk to transition to a psychotic disorder. This suggests a common etiology across clinical and non-clinical individuals along a multidimensional psychosis continuum that may be detectable in structural variations of the brain. The current diffusion tensor imaging study assessed 50 healthy individuals (35 females) to identify possible differences in white matter associated with hallucination proneness (HP). This approach circumvents potential confounds related to medication, hospitalization, and disease progression common in clinical individuals. We determined how HP relates to white matter structure in selected association, commissural, and projection fiber pathways putatively linked to psychosis. Increased HP was associated with enhanced fractional anisotropy (FA) in the right uncinate fasciculus, the right anterior and posterior arcuate fasciculus, and the corpus callosum. These findings support the notion of a psychosis continuum, providing first evidence of structural white matter variability associated with HP in healthy individuals. Furthermore, alterations in the targeted pathways likely indicate an association between HP-related structural variations and the putative salience and attention mechanisms that these pathways subserve.

## 1 Introduction

Hallucinations are externally attributed percepts in the absence of corresponding sensory input (Bentall, 1990). They are common in multiple psychiatric and neurological conditions but also occur in the general population (Reininghaus et al., 2016, Rollins et al., 2019, Van Os et al., 2000, Waters and Fernyhough, 2017, Zhuo et al., 2019). Consequently, hallucinations are considered as a contributing factor to an extended psychosis phenotype, expressed as a multidimensional continuum rather than categorical symptomatology (Healy and Cannon, 2020, Johns and Van Os, 2001, Johns, 2005, Myin-Germeys et al., 2003, Van Os, 2003). This implies that perceptions in the general population span the entire range from reliable objective experience to unreliable or non-veridical. This variance aligns with discernible degrees of individual hallucination proneness (Waters et al., 2003, Larøi and Van Der Linden, 2005).

Hallucinations occur in any sensory modality, yet auditory verbal hallucinations (AVH) are the most common with a lifetime prevalence of 6–13 % in non-clinical individuals (Beavan et al., 2011, Linscott and Van Os, 2013, McGrath et al., 2015) and have received substantial scientific interest. Among non-clinical hallucinators, who experience frequent hallucinations, higher proneness and persistence of these psychosis-like experiences increase the risk of transitioning into psychosis (Baumeister et al., 2017, Van Os et al., 2009). Shared environmental and familial risk factors in clinical and non-clinical individuals suggest that psychosis-like experiences, including hallucinations, are associated with a common etiology (Johns and Van Os, 2001, Johns, 2005, Myin-Germeys et al., 2003).

A transdiagnostic approach to psychotic experience posits common etiology for this phenomenology irrespective of diagnoses. However, the majority of neural substrates of hallucination research has been limited to psychosis patients. Functional magnetic resonance imaging (fMRI) in this clinical group provides strong evidence for miscommunication within and between functional brain networks (Alderson-Day et al., 2015, Alderson-Day et al., 2016, Ćurčić-Blake et al., 2017, Northoff, 2014). Past clinical research lacks consensus on how the expression of an etiological continuum manifests in white matter pathways connecting dispersed brain regions. This may be in part due to differing sample characteristics in clinical populations (i.e., antipsychotic medication, duration of illness, and low sample sizes, inconsistent diagnostic categories). Hence, insight into the etiology of psychosis in the general population can contribute to an understanding of the neurobiological mechanisms of hallucinations (Kelleher et al., 2010, Kelleher and Cannon, 2011, Verdoux and van Os, 2002), of which several promising candidates were identified.

One candidate pathway is the uncinate fasciculus (UF). This pathway connects the ventral temporal lobe with the inferior frontal and orbitofrontal cortex and has been implicated in processes such as memory retrieval and processing salient or emotional sensory stimuli (Iwabuchi et al., 2015, Schmahmann et al., 2008, Von Der Heide et al., 2013). It is important in conditioning the successful interaction with the external world (Olson et al., 2015). Structural abnormalities in this pathway are reported in multiple psychiatric, neurological, and developmental conditions (Von Der Heide et al., 2013). In psychosis, alterations in the UF often reveal a reduction of diffusion directionality (Kitis et al., 2012, Lei et al., 2015, Szeszko et al., 2008, Voineskos et al., 2013), in proportion to the severity of negative symptoms (Luck et al., 2011, Mandl et al., 2010, Sun et al., 2015, Szeszko et al., 2008). Conversely, studies investigating positive symptom severity, including hallucinations, have sometimes reported a positive correlation (Bopp et al., 2017, Filippi et al., 2014, Jung et al., 2020). Recent AVH theories have ascribed this variability in UF anisotropy to hypersalient processing of irrelevant stimuli or inner speech signals (Ćurčić-Blake et al., 2017). However, it remains unclear if the underlying UF white matter alterations, associated with auditory-verbal hallucinations also extend to hallucinations in any modality.

Another white matter pathway of interest is the arcuate fasciculus (AF) that connects speech-related brain areas in the posterior temporal lobe, inferior parietal lobule, and inferior frontal gyrus. The AF has been hypothesized to play a major role in auditory verbal hallucinations (Alderson-Day et al., 2015, Ćurčić-Blake et al., 2018). It has been posited that reduced communication between prefrontal speech planning regions and the auditory cortex can result in the misperception of inner speech as an external signal (Allen et al., 2007, Frith and Done, 1988, Whitford et al., 2012). DTI research in schizophrenia patients who hallucinate lends evidence to this theory, indicating reduced fractional anisotropy (FA) in the AF (De Weijer et al., 2011, De Weijer et al., 2013, Catani et al., 2011, Curčić-Blake et al., 2015, Chawla et al., 2019, Hubl et al., 2004, Leroux et al., 2017, McCarthy-Jones et al., 2015, Psomiades et al., 2016). However, there are also contradictory findings of increased FA (Hubl et al., 2004, Rotarska-Jagiela et al., 2009, Xie et al., 2019), and a positive relationship between hallucination severity and FA in the AF (Psomiades et al., 2016, Rotarska-Jagiela et al., 2009, Knöchel et al., 2012, Thomas et al., 2022) as well as the adjacent anterior SLF (Seok et al., 2007). This heterogeneity has motivated a search for associations at the level of anatomical subdivisions of the AF (Catani and De Schotten, 2008). For example, the posterior AF has been associated with dysfunctional self-other attribution of sensory input in psychosis (Eddy, 2016, Quesque and Brass, 2019).

In addition to the intra-hemsipheric association fibers abnormal inter-hemispheric communication via the corpus callosum (CC) is often reported in schizophrenia and associated with a decrease in lateralized functions ascribed to a dominant hemisphere (Ćurčić-Blake et al., 2017). This is particularly relevant for AVH, as speech and language functions typically rely on asymmetrical processing in the auditory cortices (Steinmann et al., 2014). The lateralization of these functions is affected by the degree of information transfer of the interhemispheric auditory pathway (IAP) joining left and right hemispheric homologues (Steinmann et al., 2019). Previous DTI research in psychosis reported both FA increases (Hubl et al., 2004, Mulert et al., 2012, Shergill et al., 2007) and decreases (Curčić-Blake et al., 2015, Hubl et al., 2004, Rotarska-Jagiela et al., 2009, Wigand et al., 2015, Xi et al., 2016, Zhang et al., 2018) associated with auditory hallucinations. However, both AVH (Mulert et al., 2012) and positive symptom severity (Rotarska-Jagiela et al., 2009, Whitford et al., 2010) have shown a positive correlation with FA in the CC. This heterogeneity may again be due to variability across the subdivisions of the corpus callosum.

Lastly, pathways may extend beyond cortex to include cortico-cerebellar pathways that compare the expected sensorimotor consequences of action against the reality of the sensory environment (Jordan and Rumelhart, 1992). Transformations of an action plan to expected sensory outcome (forward models) are compared to actual sensory feedback to both determine how our actions affect sensation, and how we must adapt our actions to unexpected sensory influences to reach a desired outcome (Wolpert et al., 2011). It has been hypothesized that a sense of agency emerges by this differentiation between the internal and external world (Friston, 2012). This perspective suggests that as a motor command is sent to the periphery, the forward model associated with the outgoing command (efference copy) is sent in parallel to the cerebellum (Tanaka et al., 2020, Wolpert et al., 1998). There, reafferent sensory feedback signals are continuously compared and discrepancies are communicated back via closed loops with the neocortex to inform about a need to update or adapt. It is possible that hallucinatory experience emerges from a breakdown in this system along the pathway of cortical efference copy signaling, or via cerebellar error signaling (Pinheiro et al., 2020).

This existing evidence for white matter variability demonstrates in almost all cases decreased FA for psychosis patients who hallucinate compared to non-clinical controls (Rotarska-Jagiela et al., 2009, De Weijer et al., 2011, De Weijer et al., 2013, Zhang et al., 2018, Di Biase et al., 2020, Thomas et al., 2022). However, this pattern may not be specific to hallucinations as suggested by decreased FA in persons with schizophrenia (non-specific to hallucinatory symptoms) compared to otherwise healthy individuals (Karlsgodt, 2016, Kelly et al., 2018, Zhao et al., 2022). To address this potential confound, some research includes a non-hallucinating psychosis control group. The pattern of decreased FA in hallucinating patients relative to non-clinical controls is often repeated, however, comparisons in those studies to non-hallucinating patients provide conflicting results. This includes decreased (Hubl et al., 2004, McCarthy-Jones et al., 2015, Wigand et al., 2015, Oestreich et al., 2016, Chawla et al., 2019, Chawla et al., 2022), increased (Hubl et al., 2004, Seok et al., 2007; Wang et al., 2021), and no significant difference in FA for hallucinating relative to non-hallucinating patients (Catani et al., 2011, Leroux et al., 2017). Accumulating evidence for a positive correlation of diffusion directionality and hallucination is therefore perhaps more informative for white matter variability explicitly related to HP (e.g., Shergill et al., 2007, Rotarska-Jagiela et al., 2009, Knöchel et al., 2012, Psomiades et al., 2016) and AVH severity (e.g., Seok et al., 2007, Szeszko et al., 2008, Mulert et al., 2012, Bopp et al., 2017, Wang et al., 2021, Thomas et al., 2022). Although schizophrenia may lead to a decrease in anisotropy, hallucinations per se may be linked to an increase. Accordingly, we expected that such a relative increase in white matter anisotropy would be present across HP in the general population.

We examined how hallucination proneness (HP) in a non-clinical sample correlates with white matter structure in the selected tracts of interest using the Launay-Slade Hallucination Scale (LSHS) as a reliable self-report measure of HP (Larøi and Van Der Linden, 2005). For the sake of comparison with previous research, we employed region of interest (ROI) analyses of mean FA within atlas-based masks. We hypothesized that HP would be associated with greater FA in the UF, AF, CC, and cortico-cerebellar tracts.

## 2 Methods

### 2.1 Participants

Fifty-one participants were initially recruited through the SONA system and social media channels at Maastricht University, the Netherlands. All were undergraduate, graduate, or doctoral students with normal (or corrected-to-normal) hearing and vision. Participants provided informed consent and were offered university study credit for compensation. Exclusion criteria were any history of psychotic disorder, neurological impairment, metal implants, previous traumatic brain injury, claustrophobia, or pregnancy. Data from one participant was removed due to a scanning artifact. Of the remaining 50 participants (35 female), the average age was 22.52 years (SD 4.27; range 18 to 34). The study was approved by the Ethical Review Committee of the Faculty of Psychology and Neuroscience at Maastricht University (ERCPN-176_08_02_2017).

### 2.2 Hallucination proneness

To measure HP, all participants filled in the revised-LSHS (Larøi and Van Der Linden, 2005). This five-point Likert scale self-report questionnaire consists of 16 questions, with items targeting levels of tactile, sleep-related, visual, and auditory hallucinations as well as vivid thoughts and daydreaming (questionnaire descriptive statistics listed in Table 1). To test for covariation of these data with measures of white matter structure, we calculated total LSHS scores, comprising all 16 items for each participant as a measure of overall HP. Furthermore, we conducted an exploratory analysis on the 3 auditory items that are included in the LSHS to determine if results of white matter correlation were driven specifically by an auditory-related psychosis-like experience. This subset was previously validated as loading under a single factor through principal component analyses (Larøi et al., 2004, Larøi and Van Der Linden, 2005).

**Table 1:** LSHS Questionnaire Descriptive Statistics.

***Category***	***Items***	***μ***	***μ %***	***SD***	***Min***	***Max***	***Total***
Sleep-related	4	5.02	31.37	3.72	0	12	16
Daydreaming*	4	6.47	40.44	3.63	0	15	16
Intrusive/Vivid thoughts*	3	5.24	43.63	3.28	0	12	12
Auditory	3	2.51	20.92	2.57	0	11	12
Visual	2	1.41	17.65	1.65	0	8	8
Total**	16	19.86	31.03	10.72	2	58	64

Table 1. Descriptive statistics of LSHS-R (Larøi et al., 2005). μ = mean, μ% = mean score divided by potential maximum, SD = standard deviation, Min = minimum, Max = maximum, Total = potential maximum, * = shared item (“Sometimes my thoughts seem as real as actual events”), ** = includes item not in category (“In the past, I have smelt a particular odor when there was nothing there.”)

### 2.3 Data acquisition

Neuroimaging data were collected using a Siemens 3 T Magnetom Prisma Fit MRI scanner equipped with a 32-channel head coil (Siemens Healthcare, Erlangen, Germany) at the Scannexus facilities (Maastricht, the Netherlands). For each participant, a T1-weighted single-shot echoplanar imaging (EPI) sequence was collected using a repetition time (TR) of 2250 ms, 2.21 ms echo-time (TE), 256 mm field of view (FoV), 192 slices interleaved, 1.0 mm slice thickness (voxel size 1.0 mm3), and anterior-posterior phase encoding direction. In the same session, diffusion weighted images were recorded using a 8400 ms TR, 53 ms TE, 204 mm FoV, 87 slices interleaved, 1.5 mm slice thickness (voxel size 1.5 mm3), parallel imaging (GRAPPA) with factor 2, 30 diffusion-encoding gradients with b-value 1000 s/mm2, one b-value 0 (no diffusion weighting), and anterior-posterior phase encoding direction. The sequence was then repeated in the reverse phase encoding direction to correct for susceptibility-induced distortion. Total acquisition time was about 13 min.

### 2.4 Data pre-processing

Imaging data were converted from DICOM to 4D NIFTI using the MRIcron software Dcm2Nii conversion tool (https://www.nitrc.org/projects/mricron/). Files containing b-value and b-vector data were retrieved simultaneously by Dcm2Nii file conversion. DTI data (pre-)processing was performed with FSL version 6.0.3 (FMRIB Software Library, Oxford, United Kingdom, https://www.fmrib.ox.ac.jk/fsl), using the FMRIB Diffusion Toolbox (FDT) (Smith et al., 2004). Topup was used to estimate the susceptibility-induced off-resonance field for both anterior-posterior and reverse phase-encode blips and to form a single corrected image (Andersson et al., 2003), non-brain tissue was then removed using the brain extraction tool (BET) (Smith, 2002), and finally eddy current-induced distortions and participant movements were corrected (Andersson and Sotiropoulos, 2016).

### 2.5 Tractography

Due to high inter-subject variability in cortico-cerebellar connection trajectory, tractography was used to model CPC and CTC fiber pathways of individual participants for mean FA extraction. In FSL FDT, crossing fibers analysis was first conducted (number of fibers = 2, weight = 1, burn in = 1000) using BEDPOSTX (Behrens et al., 2007). Using FLIRT (Jenkinson et al., 2002), all atlas-based seed and waypoint masks were registered to individual structural space using FLIRT. In the CPC pathway, tracking was conducted from each left and right M1 to the contralateral hemisphere via the pons. Conversely the CTC pathway was tracked from each left and right cerebellum via the thalamus to the contralateral M1 (details of additional waypoint and exclusion masks listed in Supplementary Table 1B). Finally, a transformation matrix was created for each dataset to convert all masks in structural space to diffusion space for tractography using linear registration in FLIRT.

Tractography was conducted using PROBTRACKX_gpu (5000 samples, 2000 steps, 0.5 step-length, 0.02 curvature threshold, 0.01 fiber threshold, 0.0 distance threshold, loop-check function applied) (Hernandez-Fernandez et al., 2019). By applying the transformation matrix, the modeled pathways were then converted back into the structural space of each participant and binarized to create masks for subsequent FA extraction.

### 2.6 Correlation analyses

The diffusion tensor was fitted to the images with DTIFIT, characterizing diffusivity such as FA and mean diffusivity (MD) (Behrens et al., 2003). Additionally, axial diffusivity (AD) and radial diffusivity (RD) were calculated manually from the output (Alexander et al., 2007). FA is a measure of the proportional magnitude of directional movement of water along axonal fibers, which is commonly referred to as an indicator of white matter diffusivity (Basser, 1995). It may be enhanced by various factors such as increases of parallel diffusion, restriction of perpendicular diffusion, or a combination of these aspects. Moreover, these detectable changes to diffusion are susceptible to the contribution of multiple physiological variations such as in myelination, axon density, or membrane permeability (Jones et al., 2013). Therefore, in areas of significant FA differences, measures of MD, AD, and RD representing the magnitude of water diffusion over all directions, parallel, and perpendicular to the tract can help inform the neurobiological interpretation of FA (Alexander et al., 2007). Finally, the tract-based spatial statistics (TBSS) toolbox was used to erode remaining non-brain tissue, register, and warp all participants images to a common space, and produce a mean FA skeleton (Smith et al., 2006). This process was repeated with the non-FA TBSS function to produce whole-brain maps of mean MD, AD, and RD.

**Fig. 1:**
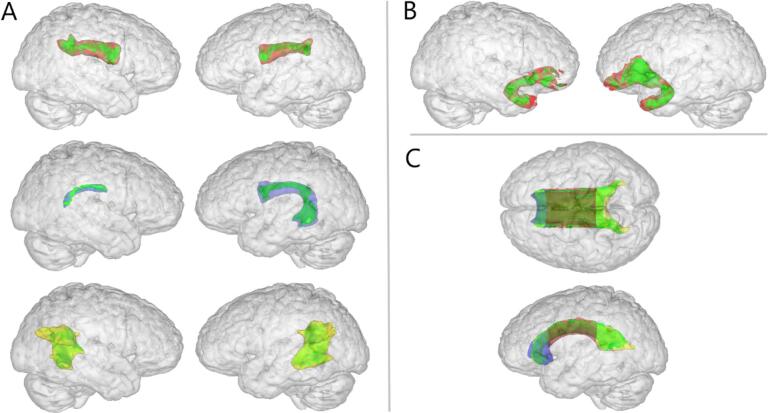
Region of interest white matter pathways. Green = voxels from group mean fractional anisotropy skeleton used in the correlation analysis; A. NATRAINLAB atlas Perisylvian network (arcuate fasciculus) masks^52^: red = anterior segment, blue = longitudinal segment, yellow = posterior segment; B. red = JHU-white matter tractography atlas uncinate fasciculus masks; C. ICBM-DTI-81 white matter atlas corpus callosum: red = body, blue = genus, yellow = splenium.

Atlas-based masks were multiplied with the mean FA skeleton mask and re-sampled to match FMRIB58 FA 1 mm space (Supplementary Table 1A): These included UF spanning anterior temporal, left and right anterior, longitudinal, and posterior AF (Fig. 1A), left and right UF (Fig. 1B), and body, genu, and splenium of the CC (Fig. 1C). Analysis in CPC and CTC tractography-modeled masks were carried out in individual diffusion space. Bivariate Pearson correlation analyses between mean FA and total LSHS were conducted for each ROI (IBM SPSS Version 26). To account for multiple comparisons, a false discovery rate (FDR) Benjamini-Hochberg correction was applied with an adjusted alpha of 0.05.

## 3 Results

### 3.1 Atlas-based correlation analyses

**Fig. 2:**
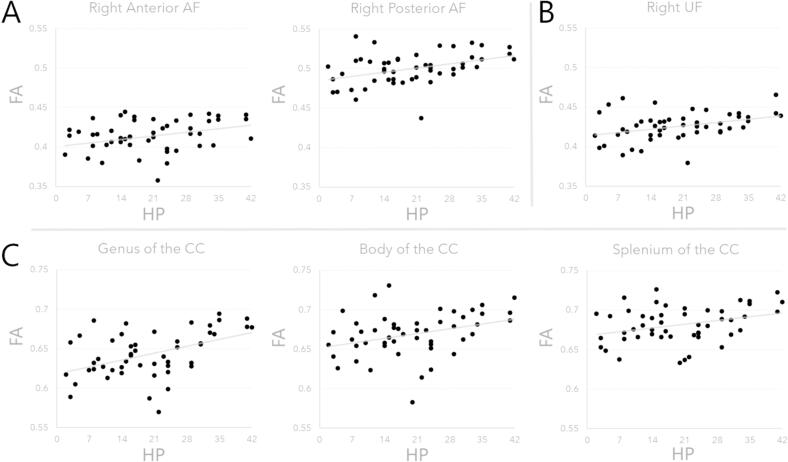
Atlas-based ROI correlation results. Pearson’s correlation between mean fractional anisotropy and LSHS composite score of hallucination proneness. A. Arcuate fasciculus: right anterior (*r*_(49)_ = 0.327, FDR-*p* = 0.042) and right posterior segments (*r*_(49)_ = 0.401, FDR-*p* = 0.021); B. Right Uncinate fasciculus (*r*_(49)_ = 0.362, FDR-*p* = 0.036); C. Corpus callosum: genus (*r*_(49)_ = 0.457, FDR-*p* = 0.009), body (*r*_(49)_ = 0.331, FDR-*p* = 0.042), splenium (*r*_(49)_ = 0.321, FDR-*p* = 0.042).

The distribution of HP scores in the sample was not significantly different from the normal distribution (p = 0.078). However, as the test of normality neared rejection, a bootstrapping procedure (1000 samples) and confidence interval correction was adopted for all correlation analyses between white matter measures and HP. Significant positive correlations between LSHS hallucination proneness scores and FA were found in the right anterior and posterior portions of the AF [Table 2A, Fig. 2A], as well as in the right UF [Table 2B, Fig. 2B]. Likewise, all subsections of the CC (genu, body, and splenium) displayed a positive correlation with hallucination proneness scores [Table 2C, Fig. 2C].

**Table 2:** Atlas-Based Roi Correlation Results.

**Tract**		***r***	**95 % CI (bias-corrected)**	**FDR*-p***
A. **Arcuate Fasciculus**				
**Left**	**Longitudinal**	0.218	0.005–0.435	0.156
	**Anterior**	0.186	−0.064–0.425	0.196
	**Posterior**	0.261	−0.007–0.490	0.092
**Right**	**Longitudinal**	0.197	−0.045–0.428	0.187
	**Anterior**	0.327	0.051–0.567	0.042*
	**Posterior**	0.401	0.119–0.672	0.021*
B. **Uncinate Fasciculus**				
**Left**		0.297	0.044–0.523	0.057
**Right**		0.362	0.026–0.665	0.036*
C. **Corpus Callosum**				
**Genus**		0.457	0.139–0.704	0.009*
**Body**		0.331	0.079–0.586	0.042*
**Splenium**		0.321	0.022–0.582	0.042*

Table 2. Pearson’s correlation analysis results between mean fractional anisotropy and LSHS composite score of hallucination proneness. Benjamini-Hochberg FDR-*p* correction for multiple comparisons (* = *p* < 0.05 threshold for significance). Bias-corrected confidence intervals (95 %, bootstrapping with 1000 samples).

Subsequent analyses for AD, RM, and MD were conducted for pathways in which significant correlation with FA were observed. The right posterior AF and splenium and body of the CC produced a positive correlation with AD (r49 = 0.401, FDR-p = 0.012; r49 = 0.378, FDR-p = 0.020; r49 = 0.320, FDR-p = 0.036). The body of the CC also correlated negatively with RD (r49 = -0.319, FDR-p = 0.036). No atlas-based pathways reported a correlation between mean FA and the LSHS auditory item subset [Supplementary Table 2].

### 3.2 Tractography-based correlation analyses

**Fig. 3:**
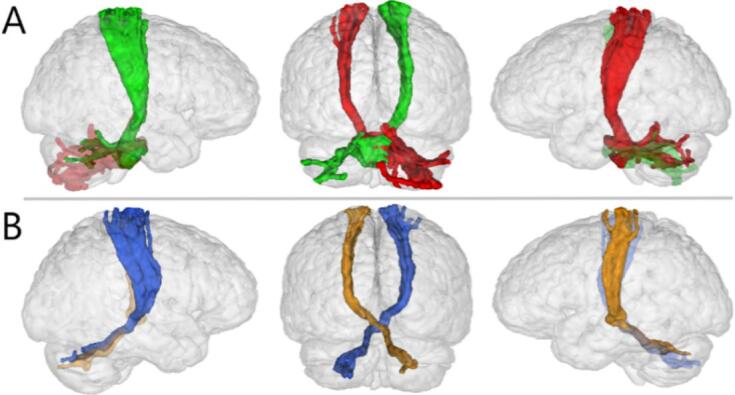
Cortico-cerebello-cortical tractography sample. A. Cortico-ponto-cerebellar pathway sample tracking (participant P08): green = right primary motor cortex to left cerebellum (221 streamlines), red = left primary motor cortex to right cerebellum (104 streamlines); B. Cerebello-thalamo-cortical pathway sample tracking (participant P08): blue = left cerebellum to right primary motor cortex (50 streamlines), orange = right cerebellum to left primary motor cortex (22 streamlines). (For interpretation of the references to colour in this figure legend, the reader is referred to the web version of this article.)

Successful tracking was completed in contralateral CPC pathways for 46 participants [Fig. 3A; Supplementary Table 1B]. In the CTC pathways, the right cerebellum to the left M1 was successfully tracked for 39 participants, and the left cerebellum and right M1 for 40 [Fig. 3B, Supplementary Table 1B]. None of the modeled cerebellar pathways revealed a significant correlation between FA and HP [Table 3], or the LSHS auditory item subset in any ROI [Supplementary Table 3B].

**Table 3:** Tractography-Based Correlation Results.

**Tract**		***r***	**95 % CI (bias-corrected)**	**FDR*-p***
**Cortico-ponto-cerebellar pathway**				
**FA**	**Right M1 → Left CE**	0.162	−0.099–0.400	0.573
	**Left M1 → Right CE**	0.128	−0.121–0.362	0.573
**Cerebello-thalamo-cortical pathway**				
**FA**	**Right CE→Left M1**	0.093	−0.189–0.358	0.573
	**Left CE→Right M1**	0.188	−0.153–0.616	0.573

Table 3. Pearson’s correlation analysis results between mean fractional anisotropy and LSHS composite score of hallucination proneness, and number of streamlines and LSHS composite score of hallucination proneness. Benjamini-Hochberg FDR-*p* correction for multiple comparisons (* = *p* < 0.05 threshold for significance). Bias-corrected confidence intervals (95 %, bootstrapping with 1000 samples).

## 4 Discussion

The experience of hallucinations by clinical and non-clinical individuals suggests a common etiology along a psychosis continuum (Johns and Van Os, 2001, Johns, 2005, Myin-Germeys et al., 2003). The current findings confirm that hallucination proneness covaries with structural white matter variability in non-clinical individuals. An increase in the directionality of diffusion was evident in right hemisphere fronto-temporal association and commissural pathways. These findings suggest that white matter pathways associated with psychosis putatively involved in salience, auditory, and sensory feedback networks are affected in individuals who are prone to hallucinate but do not require clinical care.

### 4.1 Memory/limbic networks and hypersalience

Although DTI research has indicated the involvement of UF in psychosis and psychosis-like experiences, there is no consensus regarding its relationship to phenomenology. While negative symptom severity has consistently shown a relationship with decreases in the directionality of UF diffusion (Luck et al., 2011, Mandl et al., 2010, Sun et al., 2015, Szeszko et al., 2008), positive symptoms have conversely provided some evidence for an increase (Bopp et al., 2017, Filippi et al., 2014, Jung et al., 2020). Not only has this pathway shown variability in schizophrenia, but is linked to many cognitive functions and implicated in several psychiatric, neurological, and developmental disorders (Olson et al., 2015, Von Der Heide et al., 2013). Functionally divergent subdivisions may be differentially affected, leading to heterogeneous symptoms, or may be a common contributor to transdiagnostic symptoms (Hau et al., 2016, Hau et al., 2017). Therefore, studies conducted in non-clinical groups may provide clearer findings. For example, a large sample population-based study of non-clinical adolescents with psychotic symptoms confirmed patterns of increased directionality of diffusion in the UF (O’Hanlon et al., 2015). Conversely, a decrease in the directionality of UF white matter diffusion was linked to the progression of psychosis pathology (Boos et al., 2013). This indicates divergent contributions of UF structure to symptom severity and disease progression. In line with non-clinical symptom-related findings, the current study reported FA of the right UF to be positively correlated with HP.

The UF carries internal connections of a memory and limbic system and plays an important role in the processing of salient environmental cues (Briggs et al., 2018, Hau et al., 2017, Leng et al., 2016). According to the triple-network model (Menon, 2011), erroneous engagement of the salience network can disengage the default mode network and trigger active sensory processing via the central executive network (Palaniyappan and Liddle, 2012). For example, hypersalient processing of inner speech in the default mode can result in activation of speech processing regions and the perception of external speech in the form of AVH (Alderson-Day et al., 2015, Alderson-Day et al., 2016, Curčić-Blake et al., 2015, Northoff, 2014). This disengagement of functional brain networks at rest is suggested to be associated with the misattribution of internally/externally generated stimuli (Robinson et al., 2016). Importantly, the network switching hypothesis has been related to an increased risk for psychosis or subthreshold positive symptoms including hallucinations (Bolton et al., 2020, Wotruba et al., 2014). The reported spectrum of increased directionality in white matter diffusion in the general population might therefore not only shed light on the role of the UF in salience processing, but also provide a structural region of interest for future research on the risk for developing psychosis.

### 4.2 Fronto-temporo-parietal networks top-down/bottom-up signals

The AF is commonly linked to AVH based on its putative role in self-monitoring of inner speech, during which predictions from Broca’s area are sent to the auditory cortex, leading to the suppression of cortical responses to self-generated signals (Alderson-Day et al., 2015, Allen et al., 2007, Ćurčić-Blake et al., 2018, Frith and Done, 1988, Whitford et al., 2012). However, reports of increased (Hubl et al., 2004, Rotarska-Jagiela et al., 2009) and decreased (Catani et al., 2011, Chawla et al., 2019, Curčić-Blake et al., 2015, De Weijer et al., 2011, De Weijer et al., 2013, Hubl et al., 2004, Leroux et al., 2017, McCarthy-Jones et al., 2015, Psomiades et al., 2016, Rotarska-Jagiela et al., 2009) FA in patients with AVH and variation in precise location necessitate differentiation among AF subdivisions (Catani and De Schotten, 2008). For example, some DTI studies have reported both increases and decreases in FA in separate segments of the FT pathway (Hubl et al., 2004, Rotarska-Jagiela et al., 2009, Seok et al., 2007). Furthermore, although the directionality of diffusion in schizophrenia patients with AVH provided mixed results, symptom severity of auditory hallucinations positively correlated with anisotropy (Psomiades et al., 2016, Rotarska-Jagiela et al., 2009, Seok et al., 2007). The current findings of increased FA in the AF are in line with such links to symptom severity. As the reported alterations were limited to the anterior and posterior segments of the right hemisphere, theories of left-lateralized inner-speech monitoring are not supported.

Inference models (Friston, 2005) alternatively suggest a more basic role of frontotemporo-parietal top-down predictive and bottom-up sensory input imbalance in hallucinations (Hugdahl, 2009). Due to a lack of inhibition, an influx of excitatory top-down inputs to sensory cortices results in overactivation in conditions of minimal bottom-up stimulation (Hugdahl, 2017). This can lead to misattributions of internally generated signals to an external source and an overactivation of the sensory cortices during hallucinations (Sterzer et al., 2018). Therefore, although the weak prediction signaling of self-monitoring and strong priors of predictive inference accounts differ in the proposed mechanisms, both attribute hallucinations to an overactivation of sensory cortical regions. This interpretation of increased top-down excitatory signals is in line with the current UF and AF HP-related increases in FA. Within the triple-network model, information transfer from the inferior frontal hypersalient network and central executive engagement network resulting in activation of the temporal speech regions may correspond structurally with the anterior AF segment. Indeed, the right IFG has shown increased activity in monitoring unexpected features of auditory and voice feedback (Johnson et al., 2019, Johnson et al., 2021).

The reported HP-related anisotropy increases of the right posterior AF linked to the temporal parietal junction support a broader role in a disturbed sense of agency in neuropsychiatric symptoms such as hallucinations (Eddy, 2016, Quesque and Brass, 2019). This significant positive correlation was also found for AD, a measure of diffusivity along the primary direction of diffusion (Alexander et al., 2007). As no inverse change in RD was found in the perpendicular orientations, it is possible that the overall increase in FA was associated with a change in axon density or increase in coherence of axon orientation as opposed to a decrease in myelin. However, interpretations of the underlying biophysical properties are susceptible to bias (Winklewski et al., 2018). For example, in regions susceptible to crossing-fibers where principal direction of diffusion may not reflect underlying tissue, the relationship between AD, RD, and axonal and myelin changes is less exclusive (Wheeler-Kingshott and Cercignani, 2009). The posterior portion of the AF incorporates a junction with the superior longitudinal fasciculus and may include crossing fibers. A further limitation of the current results is that the reported atlas-based parcellations of the AF contain some overlap between the identified subregions. Therefore, although the longitudinal AF subsegment did not show a significant difference, we cannot rule out if longitudinal association fibers joining IFG to STG are included across anterior and posterior AF ROIs.

### 4.3 Interhemispheric miscommunication and hemispheric specialization

Contrary to theories of IAP involvement, the current study and previous AVH investigations show that variability in the anisotropy of CC commissural pathways is found across different subdivisions. The current findings indicate that the genu, body, and splenium are susceptible to greater anisotropy with increasing HP. Additionally, a positive correlation between HP and AD was found in the body and splenium, and a negative correlation to RD in the body. Due to the uniformity of the commissural fibers across the CC, it might be surprising that the biophysical contributions to FA correlation were not ubiquitous. However, although variability in FA of the human CC is mostly linked to axonal density, the posterior body linking precentral areas displays a link to myelination consistent with the localization of our negative correlation to RD (Friedrich et al., 2020).

Two opposing theories have been proposed to understand how CC structure might affect hemispheric specialization in regions affected by AVH (Leroux et al., 2015). In line with the current findings of increased FA of the right AF in the typically left-specialized language network, the excitatory model postulates that increased interhemispheric information transfer decreases hemispheric specialization (Bloom and Hynd, 2005). Conversely, interhemispheric miscommunication theory (IMT) suggests an inhibitory model where decreased interhemispheric communication maintains independent processing between the hemispheres (Steinmann et al., 2019). An extension of the IMT proposed that top-down prefrontal cognitive control is preserved in non-clinical hallucinators (Hugdahl, 2009). This allows for a perceptual experience of hallucination without the belief that the voice comes from an external source. Therefore, this account is incongruent with the current proposals that increasing HP is associated with top-down cognitive control. However, the increased FA in the genus of the CC that carries interhemispheric connections between the frontal lobes may support a compensatory role of right-specialized prefrontal cognitive control in non-clinical HP to counteract decreased specialization of the left-specialized language network.

### 4.4 Cortico-cerebello-cortical loops and sensorimotor feedback error

The cerebellar role in sensorimotor feedback processing provides another key element in the phenomenology of hallucinatory experience (Pinheiro et al., 2020, Tanaka et al., 2020, Wolpert et al., 1998). As in models of perceptual inference, the proposed cerebellar mechanisms rely on prediction (Friston and Herreros, 2016). However, the function of this system is to facilitate successful interaction with the environment, and therefore requires actual sensory feedback for learning and updating forward models of expected sensory outcomes of action (Miall et al., 1993). Cortico-cerebellar-cortical circuits form closed-loops, where efference copies are propagated to the cerebellum via the pontine nuclei, compared to actual reafferent feedback, and resulting discrepancies are then signaled back to the cortex via the thalamus (Welniarz et al., 2021). Altered functioning of this system can result in the loss of distinction between internally and externally generated sensation (Pinheiro et al., 2020). However, very few studies have attempted to link hallucinations or the severity of positive psychosis symptoms to the structure of cerebellar pathways (Filippi et al., 2014, Kim et al., 2014, Zhang et al., 2016). Moreover, these studies were conducted exclusively in patient samples and employed varied methodologies that produced inconsistent results.

Using tractography, we modeled and analyzed the cortico-cerebellar-cortical loop between motor cortex and cerebellar cortex of each participant [Fig. 3]. Results indicated no significant correlation of FA with measures of HP. This negative result might be explained by methodological aspects. In a *meta*-analysis that incorporated morphological, diffusion MRI, and functional connectivity (Pinheiro et al., 2021), the localization of cerebellar dysfunction was more strongly attributable to AVH symptomatology than the presence of positive symptoms in general. Although the LSHS provides reliable measures of HP as well as a subset of auditory items, the current participant group did not include persons who reported an increased sense of hearing voices. It is therefore possible that cerebellar pathway differences are only discernible in clinical manifestations where AVH frequency and severity are high. In addition, variability in the CPC/CTC tract-building might hinder the interpretation of FA results across the current group. In a small number of participants, tracts were not successfully modeled, reducing the sample size for the cerebellar pathway analyses. For those participants where pathways were successfully modeled, the number of streamlines across participants was inconsistent, suggesting variability in tractography outcomes. It is possible that, unlike the cortico-cortical ROIs where location and number of contributing voxels are standardized across participants, mean FA is not an adequate measure to probe CPC/CTC differentiation. Therefore, we suggest that future research should focus on white matter axon microstructure contributions to cerebellar tractography differences.

### 4.5 Future directions

In the current study, we adopted DTI analysis to provide general population findings with methods comparable to existing schizophrenia research. Using FA, it can be inferred that microstructural variability has influenced the overall dispersion of water through regions of white matter. Although inclusions of RD, MD, and AD allow for additional inferences to be drawn regarding the contribution of myelin and axonal differences (Alexander et al., 2007), other methods must be used to characterize and quantify further aspects of white matter microstructure. For example, neurite orientation dispersion and density imaging (NODDI) has been applied in various patient groups, including schizophrenia, to assess white matter variability across diagnostic spectra, compartmentalizing diffusion of intracellular and extracellular tissue microenvironments, and extracellular free-water (Kraguljac et al., 2023).

## 5 Conclusions

Hallucinations are a transdiagnostic phenomenon in multiple disorders and the general population. The results of the current study support an etiological continuum model of psychosis, as white matter pathways involved in salience (UF), perceptual inference (AF), and interhemispheric communication (CC) increase in the directionality of diffusion as the proneness to hallucinate rises in healthy individuals. All significant FA differences were found along the midline in the corpus callosum or limited to the right hemisphere, further suggesting variation in the lateralization of pathways in those more likely to hallucinate. Despite careful modelling of difficult to study cortico-cerebellar-cortical sensorimotor feedback loops of the human brain, FA in these pathways did not correlate with HP. As hallucinatory experience is seen in different patient groups and the general population, we attempted to outline the putative roles of the affected white matter pathways in generalized brain mechanisms, as opposed to deficits reported in clinical groups. The current evidence suggests that variability in white matter structure associated with proneness to hallucinate may be related to mechanisms responsible for the attribution of salience and attention to sensory inputs, and not specific to language networks. The current findings support the contribution of a common etiology for HP across a continuum. We suggest that further research should reveal how this variability in structure may indicate a potential risk to transition to illness.

## Disclosures

All authors (Joseph F. Johnson, Michel Belyk, Michael Schwartze, Ana P. Pinheiro, and Sonja A. Kotz) declare no potential sources of conflict of interest.

## CRediT authorship contribution statement

**Joseph F. Johnson:** Conceptualization, Formal analysis, Investigation, Writing – original draft, Writing – review & editing. **Michael Schwartze:** Conceptualization, Supervision, Writing – review & editing. **Michel Belyk:** Conceptualization, Methodology, Supervision, Writing – original draft, Writing – review & editing. **Ana P. Pinheiro:** Conceptualization, Funding acquisition, Supervision, Writing – review & editing. **Sonja A. Kotz:** Conceptualization, Funding acquisition, Investigation, Resources, Supervision, Writing – original draft, Writing – review & editing.

## Declaration of competing interest

The authors declare that they have no known competing financial interests or personal relationships that could have appeared to influence the work reported in this paper.

## Data Availability

Data will be made available on request.
