# Supplementary material for: Variability in white matter structure relates to hallucination proneness
Source: Neuroimage Clin. 2024 Jul 20;43:103643. doi: 10.1016/j.nicl.2024.103643 (PMC11325372; doi:10.1016/j.nicl.2024.103643)
Supplement: Supplementary Data 1 [file mmc1.docx]

**Supplementary Information**

**Supplementary table 1. Mask details**

| ***A. Atlas-based ROI masks*** | | | |
| --- | --- | --- | --- |
| **Pathway** | | | **Atlas** |
| Uncinate Fasciculus (L/R) | | | JHU-WM tractography |
| Arcuate Fasciculus (L/R) | | Anterior | NATRAINLAB |
|  |  | Longitudinal |  |
|  |  | Posterior |  |
| Corpus Callosum | | Body | JHU ICBM-DTI-81 WM |
|  |  | Genus |  |
|  |  | Splenium |  |
| ***B. Atlas-based tractography-guiding masks*** | | | |
| **Pathway** | **Mask type** | **Region** | **Atlas** |
| Cortico-thalamo-cortical pathway | Seed | Cerebellum (L/R) | FSL Cerebellar Atlas in MNI152 |
|  | Inclusion (waypoints) | Superior cerebellar peduncle (IL) | JHU ICBM-DTI-81 WM |
|  |  | Ventrolateral thalamic nuclei (CL) | Talaraich Daemon |
|  | Exclusion | Middle cerebellar peduncle | JHU ICBM-DTI-81 WM |
| Cortico-ponto-cortical pathway | Seed | Primary Motor (L/R) | Julich Histological |
|  | Inclusion (waypoints) | Cerebral peduncle* (IL) | JHU ICBM-DTI-81 WM |
|  |  | Pons | Talaraich Daemon |
|  |  | Middle cerebellar peduncle (CL) | JHU ICBM-DTI-81 WM |
|  |  | Cerebellum (CL) | FSL Cerebellar Atlas in MNI152 |
|  | Exclusion | Cerebellum (IL) |  |
| **Sup. Table 1.** R = right, L = left, CL = contralateral, IL = ipsilateral. *Manual restriction to posterior corticopontine segment of the crus cerebri, to avoid tracking both parallel corticospinal fibres coming from the same region of M1 and other corticopontine fibres. | | | |

**Supplementary table 2. Atlas-based ROI LSHS-auditory sub-score correlation results**

| **Tract** | | ***R*** | **95% CI (bias-corrected)** | **FDR-p** |
| --- | --- | --- | --- | --- |
| ***Arcuate Fasciculus*** | | | | |
| Left | Longitudinal | 0.120 | -0.129-0.415 | 0.457 |
|  | Anterior | 0.153 | -0.066-0.422 | 0.387 |
|  | Posterior | 0.197 | -0.041-0.409 | 0.267 |
| Right | Longitudinal | 0.047 | -0.188-0.303 | 0.745 |
|  | Anterior | 0.202 | -0.050-0.486 | 0.267 |
|  | Posterior | 0.259 | 0.000 - 0.550 | 0.158 |
| ***Uncinate Fasciculus*** | | | | |
| Left | | 0.145 | -0.120-0.433 | 0.387 |
| Right | | 0.279 | 0.026-0.532 | 0.158 |
| ***Corpus callosum*** | | | | |
| Genus | | 0.260 | -0.010-0.497 | 0.158 |
| Body | | 0.350 | 0.099-0.597 | 0.143 |
| Splenium | | 0.257 | -0.005-0.495 | 0.072 |
| **Sup. Table 2.** Pearson’s correlation analysis with hallucination proneness LSHS-R auditory-item subscore. FA = fractional anisotropy, # Streamlines = number of streamlines produced from tractography. Benjamini-Hochberg FDR-p correction for multiple comparisons (* = p < 0.05 threshold for significance). Bias-corrected confidence intervals (95%, bootstrapping with 1000 samples). | | | | |

**Supplementary table 3. Tractography-based correlation results**

| ***A. LSHS Total Score*** | | | | |
| --- | --- | --- | --- | --- |
| **Tract** | | **r** | **95% CI (bias-corrected)** | **FDR-p** |
| ***Cortico-ponto-cerebellar pathway*** | | | | |
| FA | Right M1 → Left CE | 0.162 | -0.099-0.400 | 0.573 |
|  | Left M1 → Right CE | 0.128 | -0.121-0.362 | 0.573 |
| # Streamlines | Right M1 → Left CE | -0.026 | -0.340-0.280 | 0.865 |
|  | Left M1 → Left CE | -0.129 | -0.363-0.137 | 0.524 |
| ***Cerebello-thalamo-cortical pathway*** | | | | |
| FA | Right CE → Left M1 | 0.093 | -0.189-0.358 | 0.573 |
|  | Left CE → Right M1 | 0.188 | -0.153-0.616 | 0.573 |
| # Streamlines | Right CE → Left M1 | 0.270 | -0.050-0.575 | 0.194 |
|  | Left CE → Right M1 | 0.420 | 0.153-0.616 | 0.028* |
| ***B. LSHS Auditory sub-score*** | | | | |
| ***Cortico-ponto-cerebellar pathway*** | | | | |
| FA | Right M1 → Left CE | 0.095 | -0.206-0.359 | 0.793 |
|  | Left M1 → Right CE | 0.134 | -0.163-0.393 | 0.793 |
| # Streamlines | Right M1 → Left CE | 0.055 | -0.234-0.379 | 0.718 |
|  | Left M1 → Right CE | -0.100 | -0.312-0.144 | 0.676 |
| ***Cerebello-thalamo-cortical pathway*** | | | | |
| FA | Left CE → Right M1 | -0.083 | -0.322-0.153 | 0.793 |
|  | Right CE → Left M1 | 0.043 | -0.341-0.340 | 0.793 |
| # Streamlines | Left CE → Right M1 | 0.308 | -0.051-0.618 | 0.112 |
|  | Right CE → Left M1 | 0.369 | -0.006-0.674 | 0.076 |
| **Sup. Table 3.** Pearson’s correlation analysis results between mean fractional anisotropy and LSHS score, and number of streamlines and LSHS score. Benjamini-Hochberg FDR-p correction for multiple comparisons (* = p < 0.05 threshold for significance). Bias-corrected confidence intervals (95%, bootstrapping with 1000 samples). | | | | |
